# Development and Clinical Evaluation of a Vascular Access–Optimized Perioperative Care Pathway for Temporary Pacemaker Implantation

**DOI:** 10.31083/RCM50024

**Published:** 2026-09-04

**Authors:** Huiqing Huang, Junhuan Mao, Ziguan Zhang, Zhengrong Huang, Yadan Chen, Dehua He

**Affiliations:** ^1^First Department of Geriatric Medicine, The First Affiliated Hospital of Xiamen University, School of Medicine, Xiamen University, 361005 Xiamen, Fujian, China; ^2^Nursing Department, The First Affiliated Hospital of Xiamen University, School of Medicine, Xiamen University, 361005 Xiamen, Fujian, China; ^3^Department of Cardiology, The First Affiliated Hospital of Xiamen University, School of Medicine, Xiamen University, 361005 Xiamen, Fujian, China

**Keywords:** temporary pacemaker, perioperative care pathway, median cubital vein, comfort, psychological, heart disease, nursing

## Abstract

**Background:**

This study aimed to evaluate the safety and feasibility of a perioperative care pathway for temporary pacemaker implantation using a median cubital vein approach.

**Methods:**

A total of 238 patients who underwent non-emergency temporary pacemaker implantation between September 2020 and October 2024 were enrolled and randomly assigned to either the femoral vein group (F group) or the median cubital vein group (M group). The M group received a standardized perioperative nursing care pathway tailored to median cubital vein access. Electrode dislodgement and procedure-related complications were compared between the groups. Patient comfort and emotional status were assessed using the General Comfort Questionnaire (GCQ), the Self-Rating Anxiety Scale (SAS), and the Self-Rating Depression Scale (SDS). The quality of nursing care was evaluated using predefined quality control standards.

**Results:**

The M group demonstrated significantly lower rates of electrode dislodgement and complications than the F group (*p* < 0.05). Postoperatively, GCQ scores were significantly higher in the M group, whereas SAS and SDS scores were significantly lower (all *p* < 0.05). Compliance with nursing care quality standards was also higher in the M group (*p* < 0.01). Within-group analysis showed significant reductions in SAS and SDS scores in both groups before pacemaker removal, with greater improvements observed in the M group (*p* < 0.05). No significant improvement in GCQ scores was observed in the F group. The M group showed also higher adherence to the standardized perioperative nursing care pathway than the F group (*p* < 0.01).

**Conclusion:**

Compared with femoral vein access, temporary pacemaker implantation via the median cubital vein was associated with improved safety, greater patient comfort, and better emotional outcomes. The vascular access-optimized perioperative care pathway provided meaningful clinical benefits and warrants broader implementation.

## 1. Introduction

Temporary cardiac pacemaker implantation is a widely used transitional treatment for the management of severe bradyarrhythmias, including cardiac arrest and sick sinus syndrome, and is also employed as a prophylactic measure during high-risk surgical procedures. The procedure involves advancing a pacing electrode through a venous access route into the right ventricle, where it delivers electric stimulation to maintain an adequate heart rate [1]. In clinical practice, the femoral vein and subclavian vein are the most commonly used access sites [2]. Although these vessels are favored because of their relatively large lumen, their use is associated with complications such as hematoma, pneumothorax, arteriovenous fistula, and pseudoaneurysm formation [3]. These risks highlight the need for a safer approach to temporary cardiac pacemaker implantation.

In 2020, our team successfully developed and implemented a novel technique for temporary cardiac pacemaker implantation via the median cubital vein. This superficial vein, with a diameter of approximately 4 mm, is routinely used for venipuncture, intravenous infusion, and placement of peripherally inserted central catheters (PICCs). The technique was granted a National Utility Model Patent Certificate (Patent No. ZL202022354600.1) [4]. Building on this innovation, the present study aimed to develop a standardized perioperative nursing care pathway tailored to temporary cardiac pacemaker implantation via the median cubital vein and to evaluate its clinical application and outcomes. This study was approved by the Ethics Committee of the First Affiliated Hospital of Xiamen University (Ethics Approval No.:2020038).

## 2. Patients and Methods

### 2.1 Patients

A total of 238 hospitalized patients who underwent non-emergency temporary cardiac pacemaker implantation at a single center, First Affiliated Hospital of Xiamen University, between September 2020 and October 2024 were enrolled in this study. Eligible patients were randomly assigned in a 1:1 ratio to the femoral vein group (F group) or the median cubital vein group (M group), with 119 patients in each group. The randomization sequence was generated using a computer-based random-number generator by an independent nurse who was not involved in patient recruitment or outcome assessment. Allocation concealment was ensured using sequentially numbered, opaque, sealed envelopes. Group assignment was disclosed to the attending physician only immediately prior to the procedure. Simple randomization was used, without stratification or blocking.

#### Selection Criteria

Patients aged 18–80 years who were scheduled for temporary cardiac pacemaker implantation as transitional therapy during the perioperative period were eligible for inclusion. Patients were excluded if they met any of the following criteria: (1) requirement for emergent temporary cardiac pacing therapy; (2) pregnancy or lactation; (3) presence of psychiatric disorders; (4) evident bleeding tendency or diagnosed hematological disease; (5) refusal to participate in the study.

### 2.2 Perioperative Nursing Pathway

#### 2.2.1 F Group (Femoral Vein Approach)

(1) Preoperative care: A peripheral intravenous catheter was routinely established for emergency use. The groin area was prepared, including standard skin disinfection, and patients received preoperative psychological care.

(2) Intraoperative care: Nursing staff assisted the physician with sterile preparation, draping, and local anesthesia. The femoral vein was directly punctured, and a guidewire was introduced. After withdrawing the puncture needle, a 6F vascular sheath was inserted along the guidewire. The pacing electrode was advanced via the guidewire through the femoral vein, iliac vein, and inferior vena cava to the right ventricular apex. The external cardiac pacemaker generator was connected, and pacing parameters were tested to confirm appropriate function. The pacemaker was then secured using sterile dressings, and both the exposed electrode segment and pacing device were fixed to the patient’s thigh.

(3) Postoperative care: Pacemaker function was continuously monitored to ensure stable operation of the temporary cardiac pacemaker. The puncture site was regularly inspected, and the dressing was kept clean and dry. Patients were closely observed for complications such as bleeding, infection, thrombosis, phlebitis, subcutaneous hematoma, and local ecchymosis.

(4) Positioning and activity management: Patients were prescribed bed rest following implantation. The right lateral decubitus position was avoided, and flexion of the operated limb was restricted to less than 20°. Patients received guidance on active and passive limb exercises, and their lower limbs were monitored for swelling to prevent deep vein thrombosis.

#### 2.2.2 M Group (Median Cubital Vein Approach)

(1) Preoperative care: A peripheral intravenous catheter was established for emergency use, and psychological care was provided. In addition, a 20-gauge peripheral indwelling catheter was placed in the median cubital vein, which served as the vascular access route for temporary pacemaker implantation.

(2) Intraoperative care: Nursing staff assisted with sterile disinfection and draping. The exposed portion of the 20-gauge indwelling catheter was thoroughly disinfected, after which the soft outer sleeve was trimmed. A 0.64-mm-diameter guidewire, compatible with a 6F radial artery sheath, was advanced approximately 10 cm through the outer sleeve into the vein, and the indwelling catheter was withdrawn. A vascular sheath was then introduced over the guidewire. The temporary pacing electrode was advanced through the sheath to the right ventricular apex and connected to the external pacemaker generator. After confirmation of satisfactory pacing function, the device was secured using sterile dressings, and the exposed electrode segment and pacing device were fixed to the operative arm (Fig. 1).

**Fig. 1.**
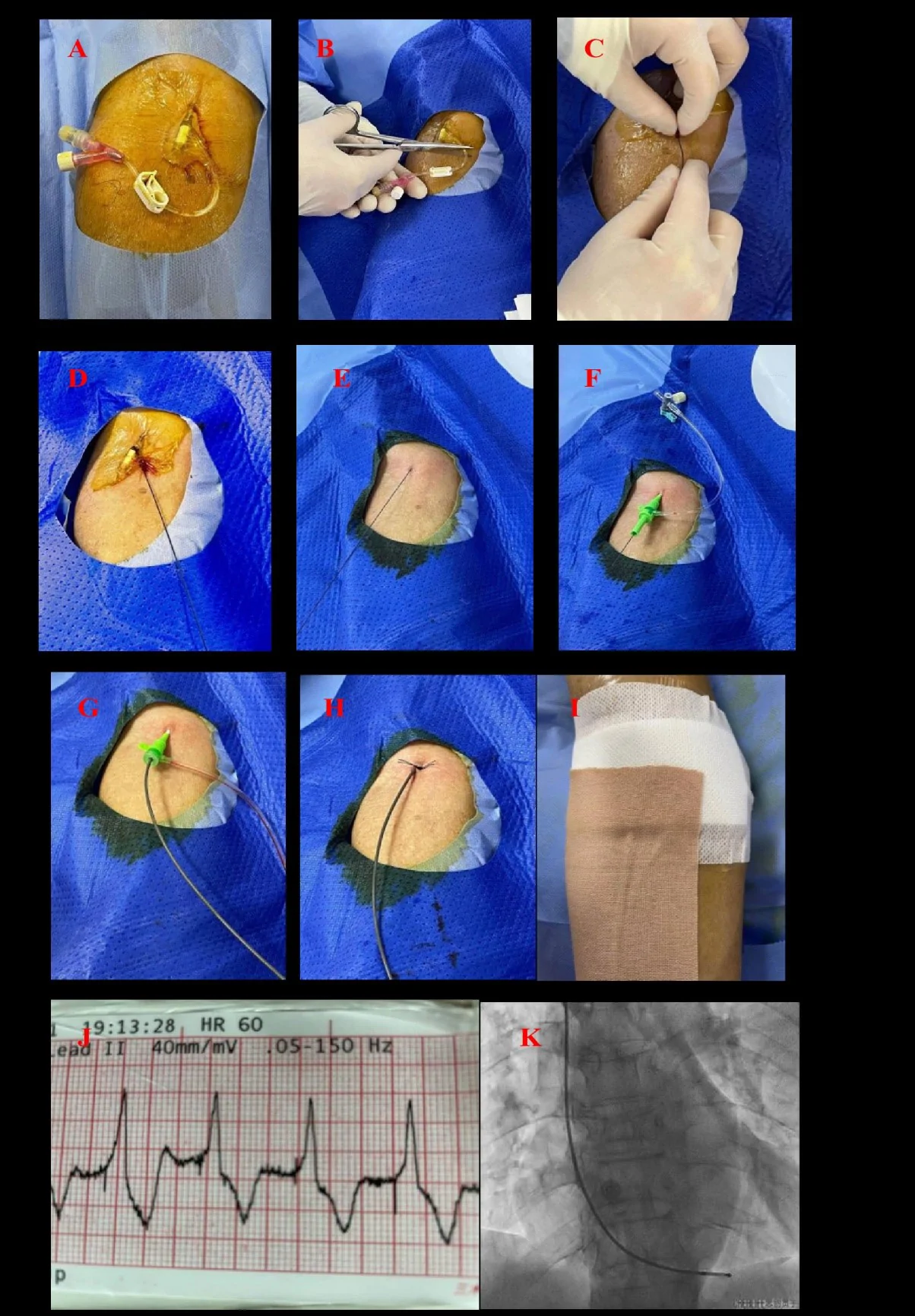
**Procedural steps of temporary pacemaker implantation procedure via the median cubital vein**. (A) Placement of a 20-gauge indwelling catheter in the median cubital vein. (B) Removal of the soft outer sleeve. (C) Insertion of the guidewire. (D,E) Withdrawal of the indwelling catheter while maintaining the guidewire *in situ.* (F) Introduction of the vascular sheath into the vein. (G–I) Advancement and fixation of the temporary pacing lead through the sheath. (J) Electrocardiographic confirmation of pacemaker function. (K) X-ray fluoroscopy to confirm the position of the pacing lead tip.

(3) Postoperative care: Pacemaker function was monitored to ensure stable operation. The puncture site was inspected regularly, dressings were kept clean and dry, and patients were observed for complications.

(4) Positioning and activity management: Postoperative bed rest or sitting was permitted. Patients were instructed to avoid the right lateral decubitus position and to refrain from vigorous arm movements, including waving, excessive abduction, or lifting with the operative arm. The lower limbs and the contralateral upper limb were permitted free movement.

### 2.3 Outcome Measures

The primary outcome was the incidence of procedure-related complications. Secondary outcomes included: (1) electrode dislodgement rate; (2) patient comfort, assessed using the General Comfort Questionnaire (GCQ); (3) anxiety and depression, measured using the Self-Rating Anxiety Scale (SAS) and Self-Rating Depression Scale (SDS); and (4) nursing care quality compliance during the temporary pacemaker retention period.

#### 2.3.1 Patient Comfort

Patient comfort was evaluated using the GCQ, a validated and widely applied self-report instrument [5]. The GCQ assesses comfort across four domains: physical, psychological, sociocultural, and environmental. Originally developed by Kolcaba in 1992, based on the Comfort Theory, the instrument was introduced in China in 2006 and has since been used in diverse clinical settings [6]. Items are rated on a four-point Likert scale, yielding total scores from 28 to 112, with higher scores reflecting greater perceived comfort.

#### 2.3.2 Mental Health Status

Patients’ mental health status was assessed using the SAS and the SDS [7]. Scores were interpreted according to standard cut-off values as follows. For SAS: >50 as depression; 50–60 as mild; 61–70 as moderate; >70 as severe (with <50 as normal).

#### 2.3.3 Procedure-Related Complications

Complications related to temporary pacemaker implantation, including thrombosis, infection, phlebitis, subcutaneous hematoma, nerve injury, local ecchymosis (diameter >1 cm), arteriovenous fistula, and pseudoaneurysm, were recorded during the indwelling period and two days after pacemaker removal.

#### 2.3.4 Quality of Nursing Care During the Temporary Pacemaker Retention Period

The quality of nursing care during the temporary pacemaker retention period was evaluated using team-developed quality control standards based on established postoperative care requirements for temporary pacemakers [8]. The assessment covered five domains: (1) dressing management at the pacemaker puncture site; (2) fixation of exposed electrodes; (3) positioning management; (4) monitoring of pacemaker parameters and clinical handover; (5) postoperative education and adherence to activity recommendations. The compliance rate (%) was calculated by dividing the number of compliant items by the total number of audited items.

### 2.4 Data Collection

Data were collected using the following approaches:

(1) Patient-reported measures: The GCQ, SAS, and SDS were self-administered by patients on the day of temporary pacemaker implantation and again prior to pacemaker removal.

(2) Nursing quality control: Quality control scores for temporary pacemaker care were assessed by the research team during morning rounds on the second postoperative day through on-site audits.

(3) Complication monitoring: Procedure-related complications were recorded daily by the attending physician in medical progress notes from implantation until 2 days after pacemaker removal. All complications were clinically diagnosed by the attending physician.

### 2.5 Statistical Methods

Statistical analyses were performed using IBM SPSS Statistics version 22.0 (IBM Corp., Armonk, NY, USA). Normality of continuous variables was assessed using the Shapiro–Wilk test. Variables with a normal distribution are represented as mean ± standard deviation. Between-group comparisons were performed using *t*-tests, and within-group comparisons (pre- vs post-procedure) were analyzed using paired *t*-tests. Categorical variables are presented as frequencies and percentages and were compared using the chi-square test. A *p*-value < 0.05 was considered statistically significant. This feasibility study did not include an a priori sample size calculation; the sample size (n = 119 per group) was based on the number of eligible patients during the study period. A post-hoc power analysis based on the observed complication rate (16.8% vs 3.4%) indicated that, with α = 0.05 and 119 patients per group, the statistical power exceeded 99%.

## 3. Results

### 3.1 Baseline Characteristics of the Study Groups

No significant between-group differences were observed with respect to baseline demographic or clinical characteristics (all *p *> 0.05; Table 1).

**Table 1.** **Comparison of baseline characteristics between the two groups**.

		F group	M group	*p*-value
		n = 119	n = 119	
Age		66.7 ± 12.9	67.5 ± 13.3	0.638
Males		67 (56.3)	72 (60.5)	0.511
BMI		22.1 ± 1.7	21.9 ± 1.8	0.379
Diagnosis	Hypertension	59 (49.6)	66 (55.5)	0.364
	Diabetes mellitus	26 (21.8)	24 (20.2)	0.750
	Coronary heart disease	53 (44.5)	58 (48.7)	0.516
	Heart failure	24 (20.2)	22 (18.5)	0.743
	Sinus arrest	14 (11.8)	16 (13.4)	0.696
	Sinus bradycardia	78 (65.5)	80 (67.2)	0.784
	AVB	27 (22.7)	23 (19.3)	0.524
Retention time (days)		3.3 ± 1.2	3.5 ± 1.3	0.219

Note: Data presented as frequency (%) or mean ± standard deviation and analyzed using the chi-square test or the independent samples *t*‐test. F group, femoral vein group; M group, median cubital vein group; BMI, body mass index; AVB, atrioventricular block.

### 3.2 Comparison of Operational Feasibility and Safety Between Groups

The incidence of electrode dislodgement was significantly higher in the F group than in the M group (*p* < 0.05; Table 2). In the F group, 20 patients experienced procedure-related complications, including local ecchymosis (10 cases, 50%), thrombosis (3 cases, 15%), hematoma (3 cases, 15%), arteriovenous fistula (3 cases, 15%), and infection (1 case, 5%). In contrast, the M group had only 4 cases of local ecchymosis (*p* < 0.05; Table 2). Bleeding complications were defined as any access-site ecchymosis or hematoma. No severe bleeding events, such as those requiring transfusion, surgical intervention, or meeting Bleeding Academic Research Consortium (BARC) ≥3 criteria, were observed. After excluding cases of local ecchymosis (10 in the F group and all 4 in the M group), the complication rate remained significantly lower in the M group (0/119, 0%) than in the F group (10/119, 8.4%; χ^2^ = 9.78, *p* = 0.002), indicating that minor bleeding events do not solely drive the observed safety advantage of the median cubital vein approach.

**Table 2.** **Comparison of operational feasibility and safety between groups**.

	F group	M group	*p* value
	n = 119	n = 119	
EDR (%)	21 (17.6)	6 (5.0)	0.002
Incidence of complications (%)	20 (16.8)	4 (3.4)	0.001

Data presented as frequency (percentage) and analyzed using the chi-square test. EDR, electrode dislocation rate.

### 3.3 Comparison of GCQ Scores Between Groups

At postoperative of implantation and prior to temporary pacemaker removal, patients in the M group had significantly higher GCQ scores (60.5 ± 7.5 and 62.7 ± 7.1, respectively) than those in the F group (55.7 ± 6.8 and 54.3 ± 7.3, respectively), with a mean difference of 4.8 (95% CI, 2.98 to 6.62; *p* < 0.05) and 8.4 (95% CI, 6.57 to 10.23; *p* < 0.05), respectively. There was a significant improvement in GCQ scores in the M group with a mean difference of 2.2 (95% CI, 1.03 to 3.37; *p* < 0.05). In contrast, improvements in GCQ scores in the F group did not reach statistical significance (*p* > 0.05; Fig. 2).

**Fig. 2.**
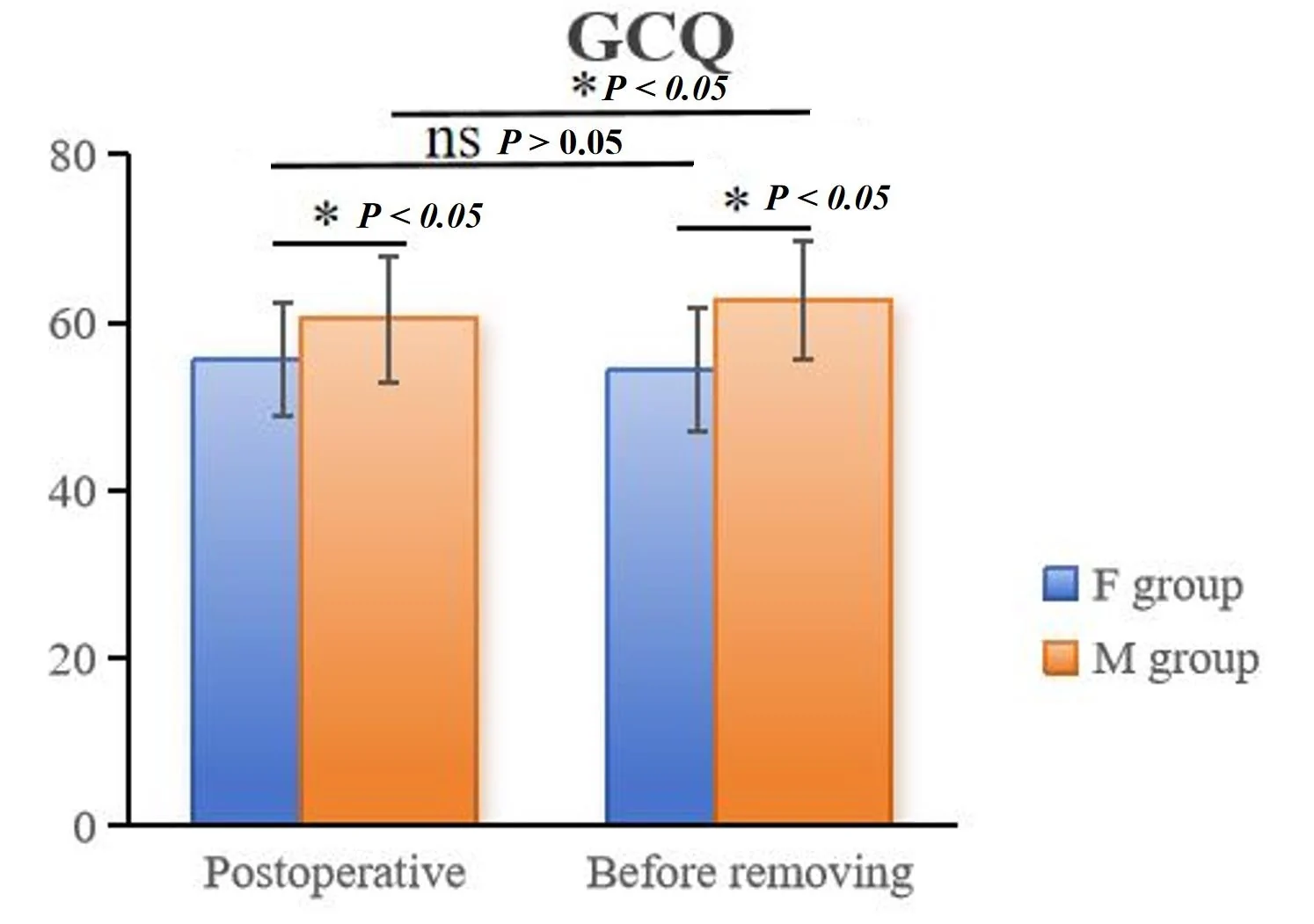
**Comparison of the General Comfort Questionnaire (GCQ) scores between the femoral vein group (F group) and the median cubital vein group (M group) at postoperative of pacemaker implantation and prior to temporary pacemaker removal**. Values are presented as mean ± standard deviation, with n = 119 per group. * *p* < 0.05; ns *p* > 0.05.

### 3.4 Comparison of SAS Scores Between Groups

At both assessment time points, SAS scores were significantly lower in the M group (58.5 ± 8.7 and 53.7 ± 8.5, respectively) than in the F group (61.7 ± 9.3 and 56.6 ± 8.9, respectively), with a mean difference of −3.2 (95% CI, −5.49 to −0.91; *p* < 0.05) and −2.9 (95% CI, −5.11 to −0.69; *p* < 0.05), respectively. Following optimized vascular access and standardized nursing care, SAS scores decreased significantly in both M group (from 58.5 ± 8.7 to 53.7 ± 8.5; mean difference: −4.8 [95% CI, −6.18 to −3.42; *p* < 0.05]) and F group (from 61.7 ± 9.3 to 56.6 ± 8.9; mean difference: −5.1 [95% CI, −6.56 to −3.64; *p* < 0.05]) (Fig. 3).

**Fig. 3.**
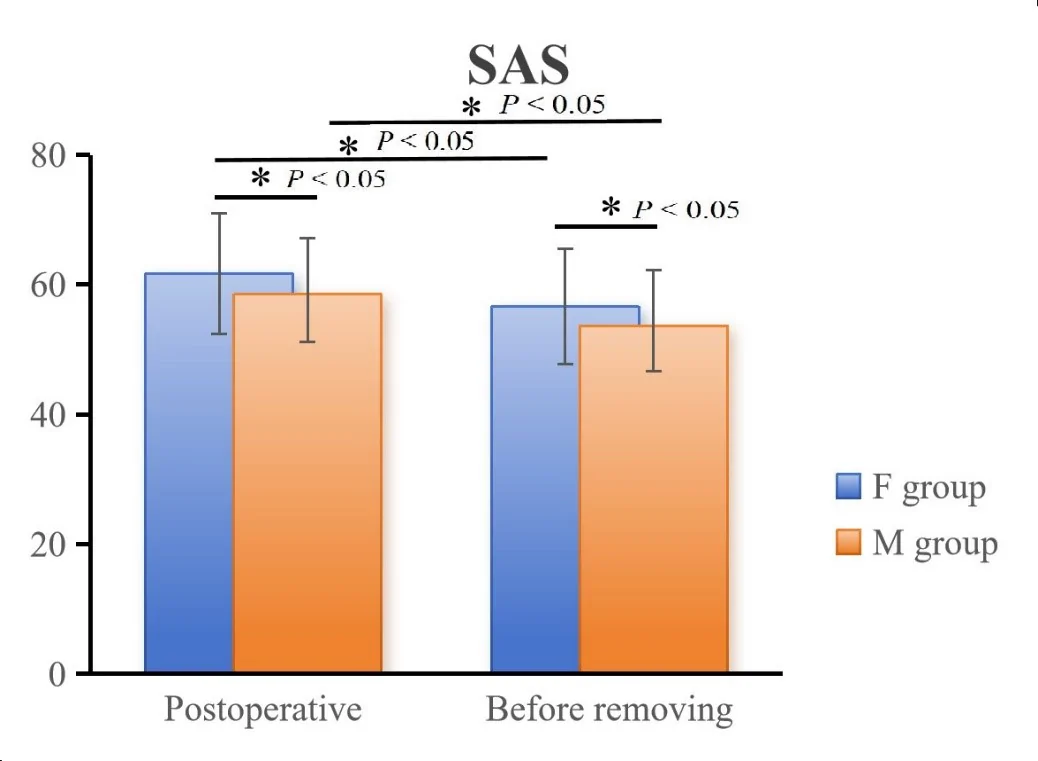
**Comparison of Self-Rating Anxiety Scale (SAS) scores between the femoral vein group (F group) and the median cubital vein group (M group) on the day of pacemaker implantation and prior to temporary pacemaker removal**. Values are presented as mean ± standard deviation, with n = 119 per group. * *p* < 0.05.

### 3.5 Comparison of SDS Scores Between Groups

SDS scores were significantly lower in the M group (55.2 ± 8.5 and 50.9 ± 8.3, respectively) than in the F group (59.3 ± 8.9 and 55.7 ± 8.6, respectively) on the day of implantation and prior to pacemaker removal, with a mean difference of −4.1 (95% CI, −6.31 to −1.89; *p* < 0.05) and −4.8 (95% CI, −6.95 to −2.65; *p* < 0.05), respectively. Standardized nursing strategies combined with optimized vascular access were associated with a further significant reduction in SDS scores in both M group (from 55.2 ± 8.5 to 50.9 ± 8.3, respectively; mean difference: −4.3 [95% CI, −5.65 to −2.95; *p *< 0.05]) and F group (from 59.3 ± 8.9 to 55.7 ± 8.6; mean difference: −3.6 [95% CI, −5.01 to −2.19; *p* < 0.05]) (Fig. 4).

**Fig. 4.**
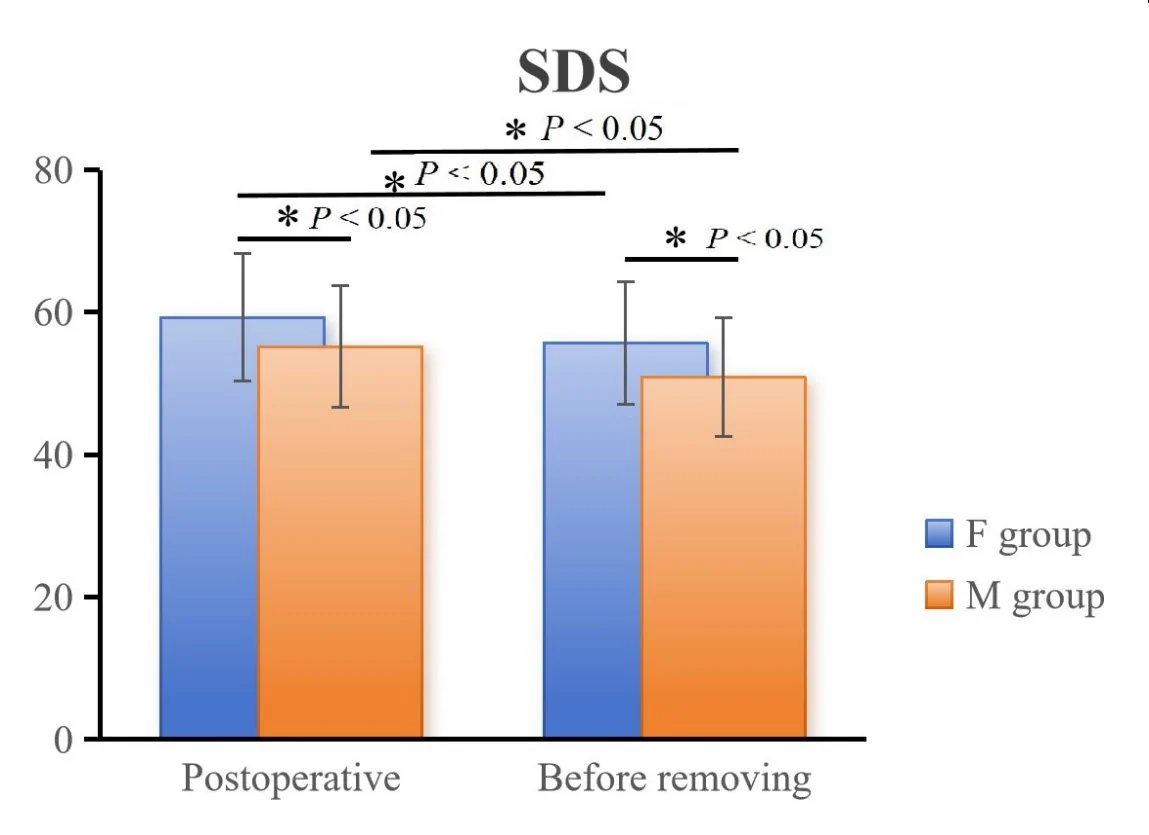
**Comparison of Self-Rating Depression Scale (SDS) scores between the femoral vein group (F group) and the median cubital vein group (M group) on the day of pacemaker implantation and prior to temporary pacemaker removal**. Values are presented as mean ± standard deviation, with n = 119 per group. * *p* < 0.05.

### 3.6 Quality of Nursing Care During the Temporary Pacemaker Retention Period

Based on the established quality control standards, the overall compliance rate was significantly higher in the M group than in the F group (86.1% vs 70.8%; χ^2^ = 41.10, *p *< 0.01; Table 3).

**Table 3.** **Compliance rates for individual nursing care quality items during temporary pacemaker retention**.

Items	Compliance rates (%)		*p* value
	F group	M group	
	n = 119	n = 119	
Dressing management at the pacemaker puncture site	86 (72.3)	92 (77.3)	0.37
Fixation of exposed electrodes	91 (76.5)	108 (90.8)	<0.01
Positioning management	75 (63.0)	105 (88.2)	<0.01
Parameter monitoring and clinical handover	109 (91.6)	113 (95.0)	0.30
Postoperative education and adherence to activity recommendations	60 (50.4)	94 (79.0)	<0.01
Overall compliance rate	70.8% (421/595)	86.1% (512/595)	<0.01

## 4. Discussion

Temporary cardiac pacing is widely used in clinical practice and can be performed through several venous access routes [9]. The femoral vein remains one of the most commonly selected access sites [1,10] because it enables rapid and technically straightforward catheter insertion. However, as a deep vein with a relatively large lumen, femoral vein access often requires repeated puncture attempts and is associated with a relatively higher incidence of procedure-related complications [10]. The reported incidence rates of complications for temporary pacemaker implantation range from 10% to 59.9% [11], with pacing lead dislodgement being the most frequently observed adverse event. In this randomized study, the median cubital vein approach was associated with a significantly lower rate of complications (3.4% vs 16.8%) and electrode dislodgement (5.0% vs 17.6%), improved patient comfort and psychological outcomes, and higher nursing care compliance.

Previous studies have suggested that temporary pacemaker dislodgement and the need for pacing lead repositioning are primarily associated with the duration of pacemaker placement rather than the vascular access route itself [12]. However, the present study showed a significantly lower rate of dislodgement with the median cubital vein approach compared to the femoral vein approach. This discrepancy may be explained by differences in patient mobility and lead traction. Pacemaker lead displacement is often related to excessive limb or body movement, which can generate mechanical traction on the externalized portion of the lead. The median cubital vein approach allows for more stable fixation and reduces lower limb movement–related traction, thereby potentially decreasing the risk of lead dislodgement.

Consistent with previous studies in patients undergoing transcatheter aortic valve replacement (TAVR), which have shown that upper-arm venous access reduces bleeding complications and facilitates earlier mobilization [13], our findings demonstrate that the median cubital vein approach significantly reduces complication rates and electrode dislodgement compared with femoral vein access. Importantly, our study extends the prior work in several ways: (1) by including a broader population of non-emergency patients requiring temporary pacing beyond the TAVR setting; (2) by providing randomized controlled evidence directly comparing median cubital and femoral vein access; and (3) by incorporating a standardized perioperative nursing care pathway with systematic assessment of patient comfort and psychological outcomes. In addition, femoral vein access required restriction of lower limb movement, leading to significant discomfort and poor adherence to postoperative activity recommendations. Only 75 of 119 patients in the F group complied with prescribed activity restrictions, a rate much lower than that in the M group. In parallel, the incidence of bleeding-related complications, such as bleeding and ecchymosis, was also higher in the F group, consistent with the findings of Chun et al. [12]. Although the femoral vein has a relatively large diameter and is anatomically predictable, its deep location and lack of direct visualization can complicate puncture, increasing the risk of vascular injury and bleeding. In contrast, the median cubital vein is superficial and readily visible, and it is routinely used for venipuncture and intravenous access, making puncture technically simpler. Postoperatively, immobilization of the forearm is easier to maintain than restriction of lower limb movement, which may contribute to improved patient comfort and a lower incidence of bleeding-related complications. Moreover, thrombosis and infection were observed exclusively in the F group. Although femoral vein access is efficient, it has gradually been replaced by the internal jugular vein approach, primarily due to the higher risk of thrombosis and other complications associated with femoral catheterization [14]. This may be related to the requirement for immobilization of the ipsilateral lower limb after femoral vein puncture to prevent catheter kinking or pacing lead displacement. Furthermore, femoral vein catheterization carries a higher risk of bloodstream infection due to potential contamination from inguinal secretions compared with other vascular access sites [15].

Patient comfort is crucial for procedural acceptance and postoperative recovery. Discomfort related to puncture sites and postoperative positional restrictions can adversely affect patient comfort [16]. In the present study, patients in the M group demonstrated significantly higher GCQ scores, which may be attributed to fewer positional limitations and greater freedom of movement. Conversely, the femoral vein approach requires strict postoperative positioning, particularly prolonged lower limb immobilization, which substantially reduces patient comfort.

Previous studies have demonstrated that procedural discomfort is closely associated with increased anxiety and depressive symptoms, as reflected by elevated scores on standardized instruments such as the SAS and SDS [17,18]. Consistent with these findings, our results showed that both groups experienced varying degrees of postoperative anxiety and depression, with significantly higher levels observed in the F group. This pattern suggests a direct correlation between procedural discomfort and psychological distress, indicating that the greater discomfort associated with femoral vein pacing may adversely affect patients’ emotional well-being. Furthermore, SAS and SDS scores decreased in both groups prior to temporary pacemaker removal, with significantly greater improvements observed in the M group. These findings align with previous evidence indicating that patient-centered and standardized nursing interventions can effectively alleviate negative psychological responses to invasive procedures [19,20]. Notably, the use of a familiar peripheral venous access route in the M group was associated with lower levels of procedural anxiety and depression. We speculate that this may be mediated by greater psychological acceptance and adaptation; however, this hypothesis warrants formal testing in future studies.

With respect to nursing care quality, the standards developed by the research team focused on five key elements essential for maintaining pacemaker function and preventing complications: dressing management, pacing lead fixation, positioning and activity management, condition and parameter monitoring, and shift handovers [9]. The results demonstrated that the M group outperformed the F group in all measured dimensions. We speculate that the superior perioperative comfort (GCQ), along with lower SAS and SDS scores in the M group contributed to better patient compliance, which is closely linked to nursing care. The more favorable nursing compliance in the M group is consistent with the lower rates of pacing lead displacement and overall complications. In the femoral vein group, compliance with postoperative education and activity recommendations was notably low (50.4%), likely due to strict immobilization requirements (limb flexion <20° and prolonged bed rest) and associated discomfort. This low adherence may have contributed to the higher electrode dislodgement rate (17.6% vs 5.0%) and overall complication rate (16.8% vs 3.4%) in this group, potentially through patient-initiated movements causing mechanical traction on the externalized pacing lead. In clinical practice, these issues may be mitigated by adopting the median cubital vein approach when feasible, reinforcing patient education, and increasing the frequency of nursing checks to support adherence to activity restrictions.

## 5. Limitation

To assess the clinical relevance of changes in SAS and SDS scores, we applied a distribution-based method (0.5 × standard deviation) to estimate minimal clinically important difference (MCID) thresholds from our dataset. Based on the observed standard deviations (SAS: 8.7–9.3; SDS: 8.5–8.9), the estimated MCID was approximately 4.4–4.7 points for SAS and 4.3–4.5 points for SDS. The between-group differences for SDS (4.1–4.8 points) met this threshold, while those for SAS (2.9–3.2 points) were slightly below it. Within-group improvements exceeded the MCID in both groups (SAS: 4.8–5.1 points; SDS: 3.6–4.3 points). These findings suggest that the median cubital vein approach provides clinically meaningful reductions in depressive symptoms, while improvements in anxiety, although statistically significant, may be more modest. We acknowledge the absence of pacing-specific MCID thresholds as a limitation.

This study primarily included non-emergency patients; therefore, the findings should not be directly extrapolated to emergency settings, where femoral access remains reasonable and often preferred. In practice, access choice should be individualized based on urgency, operator experience, and patient anatomy. Additionally, ultrasound guidance was not used for venipuncture, as our team is highly proficient in the procedure and has not observed a reduction in puncture-related complications with its routine use. However, the lack of standardized ultrasound guidance may limit generalizability, particularly in low-volume centers or among less experienced operators. The lack of a cost-benefit analysis precludes formal evaluation of the economic implications of this approach. In addition, endpoint determination was performed by the research team rather than an independent Clinical Events Committee, which represents a further limitation. We also acknowledge that comparisons of nursing compliance between the new and standard pathways may be influenced by increased staff attention to the new approach; future cluster-randomized studies with objective compliance measures are needed. No formal a priori sample size calculation was performed, as this was a feasibility study; future multi-center studies should include prospective sample size estimation. Finally, we performed two post-hoc analyses: a sensitivity analysis excluding local ecchymosis, which supported the robustness of the safety findings, and a power analysis based on the observed complication rate (16.8% vs 3.4%), which demonstrated adequate statistical power (>99%). These findings should be confirmed in future prospective studies.

## 6. Conclusion

Temporary cardiac pacing remains an indispensable clinical intervention, yet continued efforts are required to improve procedural safety and patient comfort. Median cubital vein access is associated with fewer complications and greater patient comfort, representing a safe and advantageous alternative to the traditional femoral vein approach. This superficial venous approach simplifies temporary pacemaker implantation and makes the procedure comparable in invasiveness to routine intravenous catheterization. When combined with comprehensive and standardized nursing interventions, this approach further optimizes patient outcomes by improving comfort and reducing perioperative psychological distress. Therefore, temporary pacemaker implantation via the median cubital vein represents an optimal approach in clinical practice, meriting broader adoption. This procedure is currently being practiced at a single center, and its clinical applicability requires further validation through multicenter studies, particularly those employing a 2 × 2 factorial design, to confirm the generalizability across different medical settings and to disentangle the independent effects of access site and postoperative mobility regimen on comfort and psychological outcomes.

## Data Availability

The data of this study are available from the corresponding author upon reasonable request.
